# Supplementary material for: Combinatorial quantification of distinct neural projections from retrograde tracing
Source: Res Sq. 2023 Jan 9:rs.3.rs-2454289. Preprint. [Version 1] doi: 10.21203/rs.3.rs-2454289/v1 (PMC9882684; doi:10.21203/rs.3.rs-2454289/v1)
Supplement: 1 [file NIHPPrs2454289v1-supplement-1.pdf]

Supplementary illustration

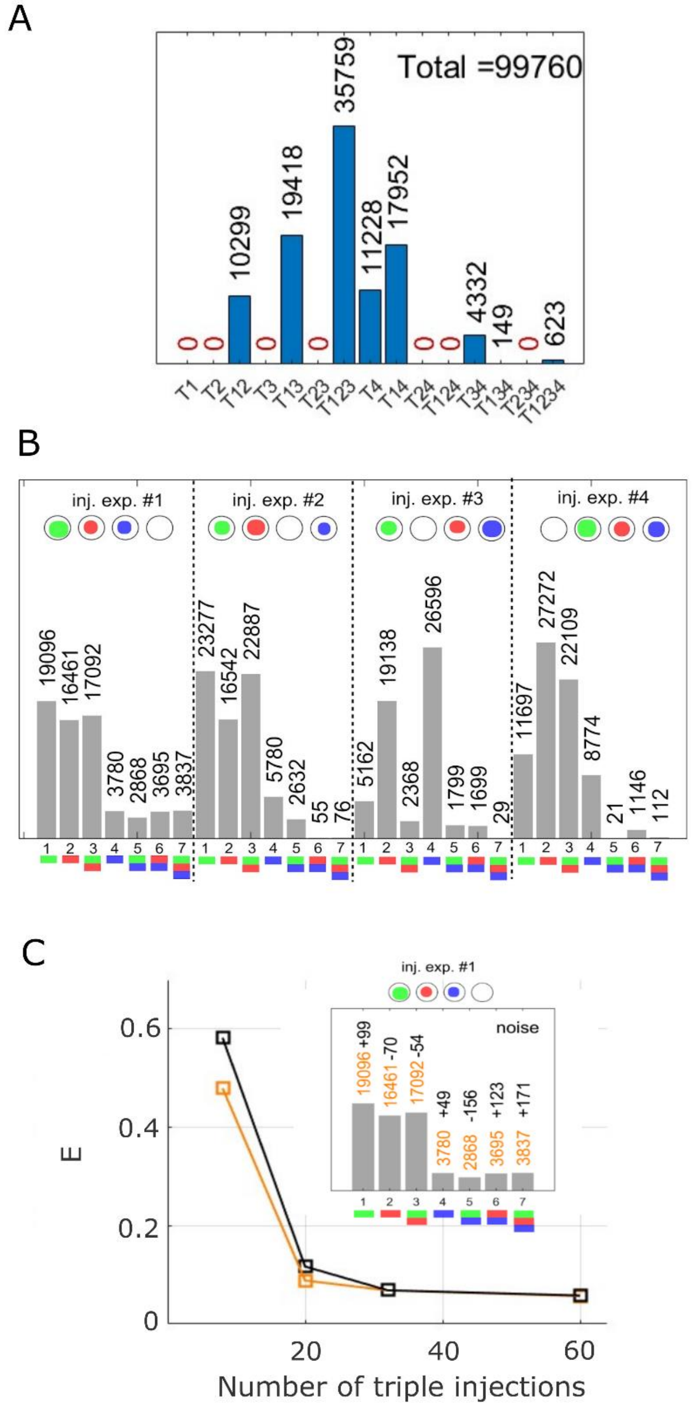

Figure S1. Analysis of $\binom{4}{3}$ model with a large total surrogate count (~100,000). **A.** Randomly generated counts for 8 projection patterns totaling 99,760. **B.** Simulated experiments with 4-triple injections based

on the surrogate counts. **C.** Error in the estimated counts by the EA for increasing number of triple injections. Black and yellow lines indicate estimation errors with and without noise respectively. See inset for an example of added noise to the constraints.

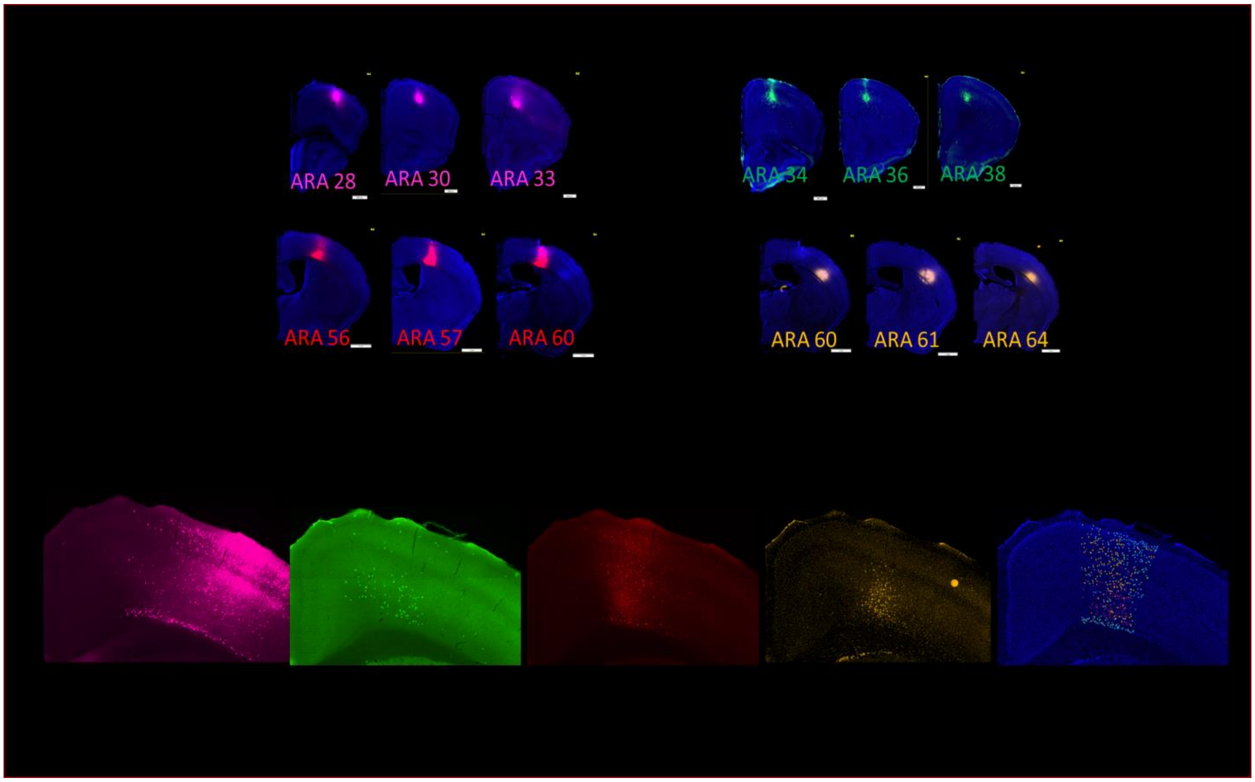

Figure S2. Infusion characteristics over 3 distinct rostro-caudal levels (top) and soma quantification (bottom) for the same experiment (and with the same color coding) illustrated in **Fig. 4.**

Supplementary Table

Table – S1. Counts of cells (constraints) expressing different color combinations in the source region

	**MO_p (total)**			**Layer 2/3**			**Layer 5**			**Layer 6**		
Repetition (R)	R1	R2	R3	R1	R2	R3	R1	R2	R3	R1	R2	R3
	515	613		266	242		149	164		158	158	
	219	38		58	34		70	3		113	2	
	17	1		3	0		10	0		2	0	
**Triple Injection #1** (MOs, MOp, SSp)	1640	1644	NA	519	595	NA	750	534	NA	521	408	NA
	91	25		16	8		23	10		50	7	
	53	1		4	1		13	0		37	0	
	4	0		1	0		1	0		2	0	
	267			130			103			92		
	19			0			0			0		
	0			0			0			0		
**Triple Injection #2** (MOs, MOp, SSs)	7	NA	NA	2	NA	NA	4	NA	NA	1	NA	NA
	22			3			11			8		
	0			0			0			0		
	0			0			0			0		
	58	104	646	2	26	299	10	21	176	48	56	134
	63	322	900	4	111	197	30	96	413	32	115	241
	2	1	71	0	0	10	0	0	42	0	1	16
**Triple Injection #3** (MOs, SSp, SSs)	18	20	174	4	2	6	1	2	24	13	14	141
	0	0	11	0	0	0	0	0	5	0	0	6
	1	9	60	0	0	0	0	1	3	0	6	50
	0	0	0	0	0	0	0	0	0	0	0	0
	288			44			109			134		
	1955			538			1056			132		
	22			3			12			1		
**Triple Injection #4** (MOp, SSp, SSs)	7	NA	NA	2	NA	NA	4	NA	NA	1	NA	NA
	3			1			0			0		
	1			0			0			0		
	1			0			0			0		

NA: Not Available

Supplementary Equations 1

Full set of equations corresponding to one triple injection experiment

$$\mathbf{G} = ((T_1 + T_{14}) * (k_1)) + ((T_{13} + T_{134}) * (k_1 * (1-k_3))) + ((T_{12} + T_{124}) * (k_1 * (1-k_2))) + ((T_{123} + T_{1234}) * (k_1 * (1-k_2) * (1-k_3)))$$

$$\mathbf{B} = ((T_3 + T_{34}) * (k_3)) + ((T_{23} + T_{234}) * (k_3 * (1-k_2))) + ((T_{13} + T_{134}) * (k_3 * (1-k_1))) + ((T_{123} + T_{1234}) * (k_3 * (1-k_1) * (1-k_2)))$$

$$\mathbf{R} = ((T_2 + T_{24}) * (k_2)) + ((T_{23} + T_{234}) * (k_2 * (1-k_3))) + ((T_{12} + T_{124}) * (k_2 * (1-k_1))) + ((T_{123} + T_{1234}) * (k_2 * (1-k_1) * (1-k_3)))$$

$$\mathbf{RB} = ((T_{23} + T_{234}) * (k_2 * k_3)) + ((T_{123} + T_{1234}) * (k_2 * k_3 * (1-k_1)))$$

$$\mathbf{GB} = ((T_{13} + T_{134}) * (k_1 * k_3)) + ((T_{123} + T_{1234}) * (k_1 * k_3 * (1-k_2)))$$

$$\mathbf{GR} = ((T_{12} + T_{124}) * (k_1 * k_2)) + ((T_{123} + T_{1234}) * (k_1 * k_2 * (1-k_3)))$$

$$\mathbf{GRB} = ((T_{123} + T_{1234}) * (k_1 * k_2 * k_3))$$
